# The involvement concept—replaceable or irreplaceable? A case for a conceptual analysis of a core concept of media psychological communication research

**DOI:** 10.3389/fpsyg.2024.1395895

**Published:** 2024-11-28

**Authors:** Holger Schramm, Zoe Olbermann, Fabian Mayer

**Affiliations:** Department of Media and Business Communication, Institute Human-Computer-Media, University of Würzburg, Würzburg, Germany

**Keywords:** involvement, ego-involvement, personal relevance, resonance, reception mode, two-process models of persuasion

## Abstract

The concept of involvement has enriched media psychological communication studies for more than three quarters of a century. The original concept of Sherif and colleagues—the so-called ego-involvement—has been extended piece by piece by various researchers over the decades, so that already in the 1980s voices were raised questioning the usefulness of such a broad meta-concept. In this article, we try to answer the question whether the involvement concept has become obsolete in the meantime by taking a more differentiated look at three different central understandings of the involvement concept (involvement as ego-involvement, as personal relevance and as mode of reception) and discussing their explanatory value. As a result, we conclude that the original conception still has its raison d’être and that media psychological communication research should take more account of this fact when designing its studies.

## 1 Introduction

Hardly any other term was and is as widespread in the research tradition of communication science across almost all sub-disciplines as involvement. For almost every reception situation, an area of application seems immediately conceivable: Consider the highly involved reception of a movie due to personal genre preferences, next to which the involved reception of a commercial promoting a product irrelevant to the individual is very low. For politically interested people, it is an absolute must to watch talk shows in the run-up to elections and to get heavily involved in them, while their possibly rather disinterested partners might sit next to them bored and without any visible emotions. The brochure of an educational organization on health issues is likely to be read by such people involved, who recognize themselves in certain symptoms and then reach directly for their smartphone to research further information. This list could be continued at will – but even now, an unpleasant feeling of conceptual ambiguity creeps in. The term “involved” in the above examples already includes cognitive, affective, and conative processes that have reference points before, during, and after the reception of the media, without really being delimited from one another.

Therefore, it is not surprising that already in the 1980s the lack of a uniform definition and conceptualization as well as inconsistent operationalization was criticized (e.g., Antil, 1984; Salmon, 1986) and voices were raised that even began to question the usefulness of such a meta-concept: “A concept that includes cognitive responses, felt emotions, attention, recall, information seeking, *and* discussions about the topic is not very useful. If involvement were all-encompassing, we could easily abandon the concept” (Wirth, 2006, pp. 209**–**210). While the frequency of use of the concept of involvement may indeed have declined somewhat in some areas of communication research in the years that followed, an analysis in the field of advertising research, for example, nevertheless shows a high prevalence of its use in published studies in the field between 2006 and 2019 (Naderer and Matthes, 2019). The authors concur with the critical conclusion of Wirth (2006), which at that time was still in the subjunctive, and, given the variety of meanings united under the umbrella term of involvement in their analysis, increase this skepticism to a call for an end to the use of the concept. In contrast, however, is the still ongoing integration of involvement chapters into the field’s textbooks and handbooks, which either strive for conceptual delineation (e.g., Bilandzic and Busselle, 2017; Bilandzic et al., 2015) or seek to gather related popular constructs of media reception research under the umbrella concept involvement (e.g., Tukachinsky and O’Connor, 2017).

The central question that arises from this will also form the core of this article: Is there a need for such a meta-concept, which, depending on the interpretation, combines many explanatory approaches and closely related concepts? Or is it not rather worthwhile to differentiate these concepts analytically in order to thereby increase the explanatory value of the various concepts and thus also of the original concept? In order to answer these questions, the historical development of the concept from its origins to its current use in numerous subdisciplines of communication science will be traced in order to then take a closer and comparative look at three different central understanding and concepts of involvement^1^ – *ego-involvement* as the original concept of involvement (Sherif and Cantril, 1947; Sherif and Sargent, 1947), *personal relevance* as central concept of high involvement in information processing (Petty et al., 1981; Zaichkowsky, 1985), and *resonance* as an involved mode of reception (Rosa, 2016, 2018; Vorderer, 2021). In the final conclusion and outlook, the three concepts are finally related to each other more closely, overlaps and differences are made clear, and we come to the conclusion that the original concept of involvement still has its raison d’être and that communication science research should take this fact more into account when designing its studies.

## 2 Conceptualizations of involvement—origins and contexts of application

The original conception of Sherif and colleagues (Sherif and Cantril, 1947; Sherif and Sargent, 1947) envisages a so-called ego-involvement as a relevant variable for understanding the reception and effect of mass media. According to this, a person’s ego consists of so-called “ego-attitudes” (Sherif and Sargent, 1947, p. 10), which represent the (never completed) result of a person’s (social) development and characterize a person’s relationship to the environment. According to this, an involved ego has a great power of influence in the medial reception situation: “In short, when the ego is involved in any situation, in any capacity, our reactions are not impartial” (p. 10). From this perspective, it is possible to understand the importance attributed to the involvement construct in subsequent decades.

Also in the mass media context, but less from a social psychological perspective and more from the perspective of advertising research, Krugman (1965) devoted himself in the 1960s to a conception of involvement to explain the effect of television advertising. He emphasizes the difference to concepts like attention, interest or excitement and defines involvement rather as so-called “bridging experiences” (p. 355), in this sense connections and references, which are produced every minute by the person watching between his own life and the stimulus. The number of these references can range from none to many, which makes it possible to distinguish between low and high involvement. To this differentiation is added a distinction regarding the involvement with the medium (television vs. print magazine) and the object (high-involvement product vs. low-involvement product) (Krugman, 1966). Authors such as Batra and Ray (1983) later join a distinction between product and message involvement but take up mainly the latter aspect and thus see involvement as a situational state, characterized by the depth and quality of the cognitive (and in their understanding also affective) reaction to a message. Accordingly, involvement is the result of many situational and interacting factors and thus not a persistent disposition.

The involvement concept also gained great importance in the 1980s with its integration into the framework of classical two-process models of persuasion. Thus, within the framework of the Elaboration-Likelihood Model, which distinguishes between deep processing on a central route and superficial processing on a peripheral route, the construct was assigned the status of a motivational variable in the sense of personal relevance (Petty and Cacioppo, 1986). The heuristic-systematic model distinguishes between systematic information processing associated with high cognitive effort and heuristic information processing associated with low cognitive effort (Chaiken, 1980). According to this model, high involvement occurs when the topics of a message are personally important to someone or when one’s own opinion to these topics is perceived as having important consequences for oneself or others.

Over the years, the involvement concept has been expanded piece by piece by different research disciplines. According to Johnson and Eagly (1989), involvement is a specific motivational state that results from a connection between an activated attitude and the self-concept. Accordingly, three aspects of self-concept can be distinguished in the influence of involvement on persuasion processes: A person’s enduring values, a person’s ability to achieve desired goals, and the impression a person makes on others. Depending on which of these aspects is activated, the three different types of involvement emerge: value-relevant, outcome-relevant, and impression-relevant, each with its own impact (see the meta-analysis by Johnson and Eagly, 1989). In the context of political communication, Rothschild and Ray (1974) added a distinction between cognitive, affective, and conative involvement to the distinction between different strengths of involvement, which has since been made by many authors (for an overview, see, e.g., Wirth, 2006, pp. 203**–**204). Salmon (1986), on the other hand, arranges findings of involvement along a continuum by means of four categories: At one end, involvement is understood as a personality trait, while at the other end it is assumed to be a stimulus property. In between lies the assumption that involvement arises from the interaction of the individual and the stimulus, with the internal state of the person playing the greater role in one case and the salience of a stimulus in the other. Levy and Windahl (1985) relate involvement to the different phases of media reception in their conceptualization of audience activity: Involvement can occur before reception (in the sense of anticipation), during reception (in the sense of attention or parasocial interaction), and after reception (in the sense of long-term identification).

In answer to the question to what extent these exemplary selections of conceptualizations are compatible with each other **–** or, more importantly, should be integrated with each other at all **–** different approaches can be found on a theoretical level: Zaichkowsky (1986), for example, combines many of the considerations in the advertising context into an inclusive model: Involvement includes the ego aspects according to Sherif and Cantril (1947) and is motivational in nature, leading to higher attention, perceived importance and corresponding behavior. Specifically, in Zaichkowsky’s understanding, personal factors (such as needs and values), stimulus factors (such as the source and content of the communication), and situational factors (such as the purchase and the opportunity) can be identified as causal for a person’s involvement. Involvement itself thus takes place at the levels of advertising, product, and purchase decision, and has a variety of consequences (such as the generation of counterarguments, preference for a particular brand, or the extent of information seeking). In contrast, approaches such as that of Bilandzic et al. (2015, p. 79) argue for a narrower understanding of involvement in order to be able to explain phenomena of reception and effect in a targeted and consistent manner in the first place. In this context, reference is made to the definition according to Donnerstag (1996, p. 31), who first states that the concept of involvement can describe a personality trait, an internal state, or situational influences, depending on the conceptualization. As a consequence, ego-involvement is narrowed down as a paraphrase for the internal commitment that people devote to a situation, topic or task to varying degrees (p. 30).

Against this background, it is hardly surprising that even within supposedly well-defined subdisciplines of communication science, there is still a clear heterogeneity in the understanding of the term, as an exemplary look at studies in the field shows: In health communication, involvement is understood on the one hand as the personal relevance of a topic (Aldoory, 2001), whereas on the other hand, parasocial and identification processes are summarized (Myrick, 2019). Elsewhere, we speak of structural features of the medium that allow a certain degree of control and require a certain type of cognitive processing (Engelberg et al., 1995). In the field of entertainment research, multidimensional operationalizations can be found, such as Hall (2009) breakdown into social, cognitive, and online involvement. In a study by Liebes and Katz (1986), on the other hand, involvement is understood as a kind of four-dimensional antithesis to detachment from the program, while Vorderer (1993) contrasts the involved, emotionally and cognitively immersive mode of reception with an analytical one. Research in the field of advertising communication is based on the one hand on the already discussed product involvement (Quester and Lim, 2003), on the other hand on the involvement with the program context in situational and cross-situational form (Tsiotsou, 2013) or investigates factors that increase the involvement with the message (Muehling et al., 1990). In political communication, for example, involvement is conceived as a multifaceted construct of knowledge, attitudes, and behaviors (Aarts and Semetko, 2003), a motivational component based on personal relevance (Heath and Douglas, 1990), or a relevance-based situational level of interest (Kushin and Yamamoto, 2010).

In summary, this small selection of theoretical conceptualizations and empirical examples already shows that the original social psychological ego-involvement has been enriched over time with processes relevant to other subdisciplines. Partly, these can now be explained more comprehensively by other established communication science and media psychology concepts (e.g., interpersonal involvement in the context of parasocial interactions, see, e.g., Hartmann et al., 2004; Klimmt et al., 2006), but partly the term involvement continues to be used for completely different concepts. The main problem seems to lie in understanding the persistence of involvement in the different concepts (*cf.* Naderer and Matthes, 2019; Wirth, 2006). The explanations of the different conceptualizations have shown: While on the one hand involvement is defined as a situation-independent person characteristic (Sherif and Cantril, 1947; Sherif and Hovland, 1961), other researchers (Petty and Cacioppo, 1986; Zaichkowsky, 1986) understand involvement as an influence factor activated by a media reception situation in the framework of two-process models. Additionally, understandings of involvement can also be found (Lombard and Ditton, 1997; Suckfüll, 2004; Vorderer, 2021) in which a specific mode of reception is described during a media reception. In the following sections of this article, these three central understandings of involvement will therefore be distinguished from each other in order to finally be able to answer the question posed in the introduction.

## 3 Understandings of the persistence of involvement

### 3.1 (Ego-)involvement as a situation-unspecific personality trait

At the beginning of this article, reference was made to the ego-involvement understanding of Sherif and colleagues. Sherif and Cantril (1947) and Sherif and Hovland (1961), respectively, first popularized the involvement concept in the social judgment theory. The theory assumes that people react differently to persuasive messages or content depending on their preconceptions: “Our reactions are considerably, and at times totally, altered according to our established or expected relationship with the individual or group in question that is, according to our roles” (Sherif and Sargent, 1947, p. 9). Preconceptions, which can strongly influence persuasive effectiveness, are formed during the lifelong development of an individual through different experiences and form a kind of anchor for the evaluation of messages. According to the researchers, they are closely linked to the recipient’s own self-image and are therefore referred to as ego-attitudes. The sum of the ego-attitudes in turn results in the ego. As soon as the ego-attitudes are activated, recipients are involved with regard to their own ego; we can speak of so-called ego-involvement, which can now influence further processing. According to the theory, people who are more involved tend to reject statements more strongly, whereas people who are less involved have relatively low latitudes of rejection but larger latitudes of acceptance or noncommitment (Sherif et al., 1965). Ego-involvement influences the persuasive effectiveness of messages especially when the content of the message is not compatible with our ego-attitudes (Sherif and Sherif, 1967). The stronger our ego attitudes are, i.e., the stronger the ego involvement, the less willing we are to accept the content of the persuasive message: “We become highly selective, accentuating certain aspects, glossing over other aspects to the point of recasting the whole situation to protect or enhance our ego” (Sherif and Sargent, 1947, p. 10). Consequently, attitude change emanating from the persuasive message is less likely, and cognitive dissonance may result (see also Carpenter, 2019). Ego-involvement, then, describes the extent to which recipients feel personally affected by an object (for example, the communicator, the topic, or the content of the message), in the sense that the object affects their self-image and sense of identity (Sherif and Cantril, 1947). The significance of ego-involvement could be empirically demonstrated even before Sherif and Cantril's (1947) theoretical elaboration. The two researchers refer to studies that consider the development and formation of the ego and its significance for the evaluation of situations already in childhood (e.g., Anderson and Brandt, 1939; Greenberg, 1932; Rosenzweig, 1933).

Based on the ego-involvement understanding of Sherif and colleagues, other researchers have dealt with it over time (e.g., Donnerstag, 1996; Johnson and Eagly, 1989; Sereno, 1968). At the beginning of this article, reference was made to Johnson and Eagly (1989), who divide involvement into three different dimensions: value-relevant involvement, impression-relevant involvement, and outcome-relevant involvement, whereby by value-relevant involvement they mean ego-involvement in the sense of Sherif and Cantril (1947). In their meta-analysis they deal with the influence of value-relevant involvement on the persuasive effectiveness of messages. In line with the considerations on ego-involvement, they assume that messages that are only slightly involving lead to stronger persuasion compared to those that are highly involving. Analysis of 15 studies confirms this hypothesis (Johnson and Eagly, 1989). The effect of value-relevant or ego-involvement can also be confirmed in more recent studies (including Choi et al., 2009; Johnson et al., 2020; Kim, 2016; Lapinski et al., 2017). Choi et al. (2009), for example, examine the impact of involvement on perceptions of the hostile media effect. Their results show that value-relevant involvement is a predictor of the perception of biased media coverage. This confirms the assumptions on the effect of ego-involvement made by Sherif and Cantril (1947) and Sherif and Hovland (1961), as it is shown that the recipient’s rejection range increases with increasing involvement and that it cannot be assumed that attitudes change in favor of the persuasive message. Lapinski et al. (2017) come to a similar conclusion. In their study, the researchers investigate the influence of value-relevant involvement on the relationship between descriptive norms and behavioral intentions in the health and environmental domain. Based on a survey, they find that involvement moderates the relationship between norms and behavioral intentions: For people with low involvement, the influence of norms on behavioral intentions was stronger compared to people with high involvement (Lapinski et al., 2017).

What insight can be gained from these explanations? The examination of the origins of involvement has shown that involvement is regarded here as ego-involvement, which describes the significance of a topic for one’s own sense of identity and is thus strongly connected with an individual’s self-concept. According to this, ego-involvement is based on attitudes that are shaped by different experiences during life and are present non-situationally. Thus, ego-involvement can be understood as a kind of situation-unspecific personal characteristic that can hardly be changed by situational circumstances but is merely activated by them.

### 3.2 Involvement as situation-(un)specific personal relevance

As already described, involvement is not understood by all scientists as a situation-independent personal characteristic. In the context of the two-process models of persuasion, involvement is seen as personal relevance that can influence the depth of elaboration of a persuasive message. Based on the two-process models, it is assumed that high involvement leads to deeper elaboration because of higher motivation (Petty and Cacioppo, 1986). Several studies tested this hypothesis in experimental settings, manipulating the relevance of the subject matter, and thus involvement. For example, Petty et al. (1981) conducted an experiment in which they manipulated participants’ personal relevance. Participants were asked to rate the introduction of a final exam in their studies, either for the following year (high relevance) or for later years (low relevance). By manipulating involvement, it becomes apparent that researchers also view involvement as a construct that is media situation-specific and thus situationally modifiable, making it different from involvement as understood by Sherif and Cantril (1947) and Sherif and Hovland (1961). Although Bilandzic et al. (2015) note that Sherif and Hovland (1961) also refer to a situation-specific form of ego-involvement, which can change, for example, during the media reception situation (also experimentally manipulated), this form of ego-involvement is not the basic understanding of the researchers.

Involvement in its meaning as personal relevance seems to be one of the most widespread understandings of the construct today. This is clearly illustrated by the operationalization of involvement in recent studies. For example, a content analysis by Naderer and Matthes (2019), which was briefly referenced at the beginning of this article, shows that Zaichkowsky (1985) scale is among the most widely used measurement tools for involvement. In its origin, the scale measures involvement regarding a product, but it is now used with regard to a wide variety of target objects (Naderer and Matthes, 2019). It is a semantic differential containing 20 item pairs, including, for example, “important/unimportant,” “interesting/uninteresting,” “boring/interesting,” or “means a lot to me/means nothing to me.” Zaichkowsky (1985) herself addresses that her measurement instrument is based on an understanding of involvement as personal relevance and describes “a person’s perceived personal relevance of the object based on inherent needs, values, and interests” (p. 342). By naming long-lasting constructs such as values, an understanding of personal relevance emerges that has parallels with situation-unspecific ego-involvement. However, further comments by Zaichkowsky (1985) show that in developing the measurement instrument, she also refers to involvement studies by Petty et al. (1981) and Petty and Cacioppo (1986), who – as just discussed – view involvement as a situation-dependent construct. Thus, it can already be stated at this point that personal relevance and involvement in the sense of ego-involvement are closely related concepts and parallels can be found, but the two are not identical. While ego-involvement is based on situation-independent attitudes, personal relevance can be both situation-independent and situation-dependent (and thus situationally changeable).

In addition to personal relevance, a number of recent studies, especially in the context of two-process models, have addressed concern and interest or knowledge about a topic as involvement (including Matthes et al., 2014; Schmuck et al., 2018; Wulf et al., 2022). This understanding is based on a definition by Hupfer and Gardner (1971), who consider involvement as a general measure of interest in a topic without reference to a specific media reception situation. This view of involvement is thus consistent with the persistence of ego-involvement, but it must be noted that knowledge or concern need not necessarily be related to a person’s ego. Thus, it seems conceivable that a person who, according to Sherif and Cantril (1947) and Sherif and Hovland (1961), is highly involved in a topic, also knows a lot about this topic and, due to current developments, can also be very concerned - but a reciprocal relationship cannot be assumed here. Or, to put it another way, high knowledge and high concern about a topic do not necessarily presuppose ego-involvement in the sense of Sherif and Cantril (1947) and Sherif and Hovland (1961).

### 3.3 Involvement as a situation-specific mode of reception

At the beginning of this article, it was already pointed out that, in addition to involvement as a personal characteristic and personal relevance, a third way of understanding the construct can be found that describes a form of involved experience, and particularly a specific mode of reception. However, it is very difficult to describe and delimit such a mode of reception in concrete terms: With regard to film reception, Vorderer (1992, 1993) sees an involved mode of reception as a specific experiential situation during film reception in which it is possible to immerse oneself in the media content, to engage with it, and to temporarily break out of reality cognitively and emotionally in order to playfully have quasi-experiences or vicarious experiences. In contrast, according to Vorderer, a distanced mode of reception is a mode of reception in which viewers are less immersed in the media content and rather analyze the making, the structure of the film, the performance of the actors or the locations and staging of scenes. Suckfüll (2004) already wondered why, however, a viewer who reflects on the production conditions of a film should be less involved, because this form of media reception can of course also lead to strong cognitive and emotional involvement - with a high probability at least among filmmakers and film experts, but sometimes also already among quite “normal” viewers who are simply fans of a certain film and are therefore interested in the story of its creation.

Also, within research on music reception, various typologies of reception modes have been designed (*cf.* e.g. Adorno, 1962; Behne, 1986; Roetter, 1987), which often distinguish between an associative-emotional devotional listening and a structural-analytical listening, but do not impute different involvement to these two forms. Quite the contrary: both forms of listening can even fuel each other and contribute to an even more intense and thus involving overall listening experience (Roetter, 1987). Involved listening is here rather opposed to modes of reception that describe “unconscious listening” (Rauhe, 1975), “inattentive or unfocused listening” (Roesing, 1985) or “diffuse listening” (Behne, 1986) and correspond to the reception situation in which someone uses music only incidentally, in the background or as musical background noise, if only to prevent silence.

To be able to weigh up the understanding of involvement as a mode of reception against the other understandings of involvement as ego-involvement or personal relevance, it is advisable to focus on the conceptions of such modes of reception that take into account the background experience of the recipient and the personal meaning of the media content for the recipient. With regard to music reception, for example, Rauhe (1975) outlines the mode of “subject-oriented reception,” which focuses on the self-knowledge of the listening subject, who finds his or her experiences, attitudes, and perceptions reflected in the music (Rauhe, 1975, pp. 138–141). With the mode of “resonance” a similar kind of involvement has been established in media reception research, which finds its use especially in the context of fictional and narrative media content. Vorderer (2021) understands resonance as a kind of eudaimonic entertainment experience (e.g., Oliver and Raney, 2011; Wirth et al., 2012), referring to Rosa (2016, 2018) understanding of resonance. Rosa (2018) describes resonance as a person’s relationship to other people, objects, or subjects that they encounter and feel addressed and touched by. This in turn leads to emotional responses from the person in the form of physiological (e.g., crying, goosebumps) and/ or reflective (e.g., pondering) responses. The extent to which a person feels touched or addressed is in turn dependent on his or her attitudes and experiences (Rosa, 2016). A high level of resonance could therefore be described as a kind of involved experience that arises from being strongly touched based on one’s own attitudes, values and experiences and that thus describes a media reception that is perceived as meaningful (Vorderer, 2021). This is precisely how resonance differs from ego-involvement, which, as already described several times, is not to be regarded as a mode of reception but, in its original understanding, as a personal characteristic that is cultivated over a long period of time and can thus hardly be changed situationally (Sherif and Cantril, 1947; Sherif and Hovland, 1961). Resonance can therefore be described as a mode of reception that arises situationally during media reception (such as personal relevance) and at the same time has references to personal experiences and values (such as ego-involvement).

At this point, another understanding of resonance should be mentioned. Resonance also appears in connection with the cultivation hypothesis (Gerbner et al., 1980) and here means the identification of parallels between the media and the real world of a person. Cultivation effects increase when a person’s attitudes shaped by experiences in the real-world match experiences on television: “When a viewer’s personal experiences involve crime and violence, heavy viewing of televisions programs depicting crime victimization may result in a ‘double dose’ of the television message and significantly boost cultivation” (Gerbner et al., 1980, p. 15). In this context, it is possible that the media content to which a person can identify parallels also relates to attitudes and values, which—as is the case with ego-involvement—relate to the person’s sense of identity and determine the person’s self-image. However, this need not be the case. As the quote from Gerbner et al. (1980) shows, resonance also occurs when the parallels concern simple experiences and observations of a person’s real life.

## 4 Conclusion and outlook

Ego-involvement, personal relevance, resonance: the examination of these three different understandings of an involved experience has shown that the constructs certainly have commonalities: All three constructs describe a strong first-person reference to the topics, content, and messages of a media offering and are therefore able to explain individual peri- and post-receptive media effects. All three assume that this first-person reference is particularly powerful because it is accompanied by a feeling of importance and significance, and this feeling results in a more attentive, conscious, elaborate and sometimes even critical processing of media content and messages, especially when the content and messages contradict one’s own convictions.

However, there are also differences between the three constructs: In the case of ego-involvement, the causal ego-attitudes have been socialized or cultivated over a longer period of time and therefore shape a person’s identity and value framework. Ego-involvement should therefore be evident during media reception primarily when topics, content, and messages address a person’s central and stable identity and value aspects or even challenge them – and these are by no means all topics, content, and messages that may seem important and significant to a person, at least in certain phases and situations. The latter would rather be well met with the construct of personal relevance, because it also shows up when the topics, contents and messages seem important and significant to the person only temporarily or only in a single moment – and be it because one’s attention was drawn to something or even manipulated directly before the media reception (e.g., to focus on a supposedly quite significant movie scene). In the sense of personal relevance, recipients are also particularly involved, for example, if they know that the contents of a media offer will be queried or tested at a later point in time (e.g., in an entrance test or an exam), or if they know that the contents could be suitable for facilitating an upcoming decision (e.g., purchase of a product). Personal relevance can thus also show up in many cases in which the identity and the value framework of a person are not affected at all. The involved experience, which comes about due to personal relevance, is thus linked to fewer preconditions and should therefore be the normal case in everyday media reception, while the involved experience in the sense of ego-involvement should represent the exceptional case.

The construct of resonance makes the differentiation even more difficult: The understanding of resonance from cultivation research is out of line and does not serve any differentiation in the sense of this article, because there any content and any message that one has already encountered in real life can trigger a form of resonance through media reception, but it is not decisive whether the content and messages are considered important and significant by the recipients. More appropriate in terms of the concept of involvement discussed in this article is therefore Rosa (2018) and Vorderer (2021) understanding of resonance as a significant and sometimes even touching experience of people, objects, or topics. Resonance arises here only in the experience, i.e., in the concrete engagement with a media content – in this respect it differs from ego-involvement, as it is not present pre-receptively as a stable person characteristic. However, according to Rosa (2016), it arises on the basis of and as a consequence of one’s own experiences, attitudes, and sometimes even values, and, according to Vorderer (2021), is only experienced as truly meaningful and sense-giving as a result. This special experience during reception has a lot in common with the exceptional case of ego-involvement outlined above, which also takes personally important attitudes and values as a basis. Resonance could therefore be understood as a mode of reception that can come about on the basis of ego-involvement. However, if one wants to find differences between these two concepts in the reception phase, one could assume that resonance should still occur when the person, the topic, or the message particularly touches one, e.g., due to personally made experiences, but without affecting the central and longer socialized identity aspects. Something can also be significant and meaningful if the significance or meaning is only recognized at the moment of reception: For example, a person who increasingly suffers from loneliness in his or her life might only recognize the meaningfulness of friendships through a film. This would be explained very well by the resonance concept, less well by the ego-involvement concept.

The distinction between all three constructs becomes even clearer with another example: According to the understanding of ego-involvement, persons are strongly involved in environmental protection if they strongly associate their attitudes toward environmental protection with their values, i.e., they also identify themselves by the attitudes they have regarding environmental protection. Involvement is part of the person regardless of whether the person is currently confronted with the environmental issue, for example, by watching an environmental documentary. This kind of involvement can also be described by personal relevance. In addition, personal relevance provides the description of another state: The personal relevance of environmental protection according to Petty et al. (1981) describes a relevance that is only activated by media reception. A person might consider environmental protection as relevant only while watching an environmental documentary due to newly gained insights – even if the person had hardly any contact with the topic before or if the topic had not touched the person’s value framework before. Such a kind of media situation-specific involvement also describes resonance according to the understanding of Vorderer (2021). Resonance in the understanding of a eudaimonic entertainment experience describes an involved experience during an environmental documentary that occurs when the person is touched by contents of the documentary based on the person’s values and experiences and thus perceives the media reception as meaningful. These values and experiences would not even have to have a direct environmental reference or a high environmental awareness: The person could be touched, for example, because the fates of affected people are shown in the environmental documentary and this activates the person’s value for a life of human coexistence, but also, for example, because a region is shown in which the person may have already spent his or her vacation.

The following table summarizes the three concepts in terms of various aspects and criteria (Table 1).

**Table 1:** Core aspects of the three different understandings of involvement.

Aspects	Ego-involvement	Personal relevance	Resonance
Core sources	Sherif and Cantril (1947), Sherif and Sargent (1947), Sherif and Hovland (1961), and Johnson and Eagly (1989)	Petty and Cacioppo (1986), Petty et al. (1981), and Zaichkowsky (1985)	Rosa (2016, 2018) and Vorderer (2021)
Definition	The extent to which recipients feel personally affected by an object (e.g., communicator, topic, content of a message), in the sense that the object affects their self-image and sense of identity (self-concept/ego-attitudes)	The extent to which the attitudinal issue under consideration is of personal importance; a person’s perceived personal importance of the object based on inherent needs, values, and interests	A kind of meaningful experience that arises from being strongly touched on the basis of one’s own attitudes, values and experiences
Individual and situational dependencies	In any case, strong individual dependencies, because ego-attitudes have been socialized or cultivated over a longer period of time; since it is a relatively stable personality trait, it can become salient in a particular reception situation and thus become more entrenched, but in principle it is more likely to be situation-independent and can also be measured independently of reception situations	Individual dependencies are not necessarily a factor, because personal importance of an issue can also be established in the reception situation, without it having been important to the person beforehand; but even in these situations, individual characteristics or circumstances can, of course, explain why some people attach more or less importance to the issue	Individual dependencies in cases in which the different significance and meaning of media content is explained by individually different characteristics of attitudes, activated values and, above all, pre-receptive experiences; depends on the fact that media content must trigger resonance in the first place, and thus on the attribution of meaning in the specific situation of reception; outside of this situation, it cannot exist
Example items	“Knowing my position on … is central to understanding the kind of person I am,” “The arguments for or against … are relevant to the core principles that guide my life.” (Cho and Boster, 2005)	Semantic differential containing 20 item pairs, including, e.g., “important/unimportant,” “interesting/uninteresting,” “boring/interesting,” or “means a lot to me/means nothing to me.” (Zaichkowsky, 1985)	If one links resonance to eudaimonic experience, one could use corresponding items: “I feel good because now that I have seen … I recognize my life as fulfilled and meaningful,” “I have a good feeling because … has made me reflect on myself and my life.” (Wirth et al., 2012)
Manipulability	No, because it is a personality trait	Yes, because the relevance can be made salient in or directly before the reception situation	Only in the sense of whether one selects stimuli for manipulation that recipients have or have not had meaningful experiences with
Practical example	Long-term and firm commitment to environmental protection	Due to media content, a short-term opinion is formed that environmental protection is important, even though the person concerned has not previously dealt with it	Due to media content, a feeling is formed that environmental protection is meaningful because it corresponds to one’s own experiences (e.g., environmental pollution)

All in all, it can thus be stated that personal relevance is easiest to establish or that it is not necessarily linked to pre-receptive conditions. Resonance in Rosa’s sense, on the other hand, requires personal experience gained prior to reception. Ego-involvement furthermore requires long-term socialization and identity formation. Personal relevance is thus the broadest concept, followed by resonance and ego-involvement. Consequently, an involved experience during media reception could always be measured with personal relevance. However, in cases where the involved experience has come about due to personal experiences or as a consequence of activated personal values and identity aspects, a relevance measurement would be too undifferentiated. As a result, the intensity of involvement, the type of involvement and any subsequent media effects could be explained less precisely. Even though the constructs can be intertwined, this article proposes a more differentiated approach. Researchers could reflect on this more in advance in future studies in order to select the appropriate concept and the appropriate measurement. What our reflections should have shown, however: The original concept of ego-involvement still has its raison d’être, because it offers its very own explanatory contribution to the processes of media reception.

## Footnotes

^1^*cf.* a first similar approach in Bilandzic et al. (2015), in the chapter “involvement, resonance and self-referencing.”
